# A survey of what legal populations believe and know about inattentional blindness and visual detection

**DOI:** 10.1371/journal.pone.0296489

**Published:** 2024-01-05

**Authors:** Hayley J. Cullen, Helen M. Paterson, Timothy S. Dutton, Celine van Golde

**Affiliations:** 1 School of Psychology, The University of Sydney, Sydney, Australia; 2 School of Psychological Sciences, The University of Newcastle, Newcastle, Australia; 3 School of Psychological Sciences, Macquarie University, Sydney, Australia; Panteion University of Social and Political Sciences, GREECE

## Abstract

Inattentional blindness refers to when people fail to notice obvious and unexpected events when their attention is elsewhere. Existing research suggests that inattentional blindness is a poorly understood concept that violates the beliefs that are commonly held by the public about vision and attention. Given that legal cases may involve individuals who may have experienced inattentional blindness, it is important to understand the beliefs legal populations and members of the community have about inattentional blindness, and their general familiarity and experience with the concept. Australian police officers (*n* = 94) and lawyers (*n* = 98), along with psychology students (*n* = 99) and community members (*n* = 100) completed a survey where they: a) stated whether an individual would have noticed an event in six legal vignettes, b) rated whether factors would make an individual more, less, or just as likely to notice an unexpected event, c) reported their familiarity with and personal experiences of inattentional blindness, and d) indicated whether they believed individuals could make themselves more likely to notice unexpected events. Respondents in all populations frequently responded “yes” to detecting the unexpected event in most legal vignettes. They also held misconceptions about some factors (expertise and threat) that would influence the noticing of unexpected events. Additionally, personal experiences with inattentional blindness were commonly reported. Finally, respondents provided strategies for what individuals can do to make themselves more likely to notice of unexpected events, despite a lack of evidence to support them. Overall, these findings provide direction for where education and training could be targeted to address misconceptions about inattentional blindness held by legal populations, which may lead to improved decision-making in legal settings.

## Introduction

It is commonly assumed that all one must do to perceive objects in the world is “look”. However, attention is a necessary requirement for perception. When individuals focus their attention on something, they may fail to notice unexpected objects or events occurring in plain sight. This experience has become known as “inattentional blindness” [1, 2]. For the most part, experiencing inattentional blindness is inconsequential and serves a productive purpose by allowing us to devote our attention to a task without distraction [3]. However, in certain contexts, failing to notice something can have disastrous consequences. For example, if witnesses fail to notice crimes [4], drivers fail to notice hazards or other road users [5], or medical professionals fail to notice abnormalities in patient scans [6], a simple failure to notice could result in serious injury or death, and legal action may follow. Thus, legal professionals will be at the forefront of some of the difficult decision-making around these failures to notice. This makes it imperative to understand the beliefs legal professionals have about inattentional blindness and visual detection.

While experiencing inattentional blindness is common, it is not intuitive [7]. Failures to notice something in our immediate field of view are treated with great surprise, as evidenced by the reactions of participants in experiments of inattentional blindness when they are later shown the unexpected object or event that they failed to notice [2, 8, 9]. Wide-scale surveys of members of the United States public also reveal that most respondents believe that individuals will notice unexpected events even if their attention is already occupied [10, 11]. Therefore, there appears to be a discrepancy between what someone would be likely to notice when focused on something else, and what others *believe* they would notice. This discrepancy suggests an overestimation of visual detection, in that people believe that someone would be more likely to notice something than the empirical research would suggest [12, 13]. In a prime example of overestimating visual detection, Levin and Angelone (2008) found that 88% of participants believed they would have noticed the gorilla in the original Simons and Chabris (1999) study of inattentional blindness when the study was described to them [12]. However, only 42% of participants noticed the gorilla in the original study [2]. Overestimations of visual detection are also common for a related phenomenon known as change blindness, such that people overestimate their ability to notice changes that take place in their visual field [12, 14, 15].

Emerging research has revealed that mistaken beliefs about inattentional blindness could have serious consequences for legal decision-making. Mock jurors who read a trial transcript depicting two witnesses—one who experienced inattentional blindness and one who noticed a crime–rated the witness who did not notice the crime less favourably than the other witness [16]. Furthermore, Jaeger and colleagues (2017) found that 26% of mock jurors who read a vignette where an individual claimed that they did not see a fight stated that the individual acted negligently. Additionally, 53% of participants who read this vignette believed that the individual had *actually* seen the assault, indicating then that this individual must have provided false testimony [17].

To determine the role that visual metacognition plays in the outcomes of legal cases, it is important to understand the beliefs that are held about inattentional blindness in legal contexts. However, the existing visual metacognition research has only drawn upon community [10, 11] or psychology student samples [12, 13]. With inattentional blindness being a fascinating demonstration of the limits of attention, it is routinely featured in introductory psychology classes. Indeed, Beanland and Pammer (2010) found that 32% of undergraduate psychology students were familiar with inattentional blindness and could describe an experiment on inattentional blindness [18]. Compared to other studies [16], the psychology students in Beanland and Pammer appear to be more familiar with the phenomenon than community members. Thus, when it comes to beliefs about inattentional blindness and visual detection, psychology students should not be relied upon to generalise to the broader community. Furthermore, while it is important to understand the beliefs about inattentional blindness held by members of the community because they will act as jurors on cases that may involve inattentional blindness, other specialist legal populations have so far been neglected from research on this topic. Therefore, it is important to extend the existing research within visual metacognition to determine what beliefs different legal populations have about inattentional blindness and visual detection.

It is crucial to understand the beliefs that police officers may have about inattentional blindness due to their interactions with witnesses. A growing body of research–both in the field and in the laboratory–has shown that it is not uncommon for witnesses to fail to notice criminal events such as thefts and physical assaults in their entirety when they are completing an effortful task [4, 7, 19–22]. If it is the case that police officers do not appreciate the possibility for witnesses to experience inattentional blindness, they may engage in inappropriate lines of questioning with these witnesses. Specifically, emerging research shows that witnesses may still answer questions about parts of an event that they did not actually notice [23], and they are likely to integrate information from other sources (e.g., misinformation) into their accounts particularly when they are pressured to respond [19]. Generally, police officers frequently ask leading or suggestive questions during interviews with witnesses [24], and leading questions are a common way in which witnesses encounter misinformation [25, 26]. As a result, if police officers do not have a good understanding of inattentional blindness, it is possible that this may affect the quality and quantity of information they collect from witnesses or victims during criminal investigations. Therefore, one aim of this survey is to gain an initial understanding of the beliefs about inattentional blindness and visual detection held by police officers, as well as their current knowledge and experiences with the phenomenon.

Lawyers may act for or against people who have claimed not to have seen an event, such as in cases involving eyewitness testimony, driving incidents, or professionals (e.g., medical professionals). Their beliefs about inattentional blindness may shape the way they prepare clients for court/legal proceedings, or the strategy they use when representing their clients. Additionally, in the absence of expert witness testimony on a given topic, lawyers will often be the people responsible for addressing misconceptions and giving jurors information to make accurate decisions [27]. Therefore, lawyers may have an important job both within and beyond the courtroom in dealing with claims of inattentional blindness, making it important to understand their beliefs about and knowledge of inattentional blindness and visual detection.

The aim of the current survey was to explore the beliefs about inattentional blindness and the detection of unexpected events held by police officers and lawyers: two legal populations that may deal with cases of inattentional blindness, but have been so far neglected from any of the research into visual metacognition. Members of the community (who may make up members of the jury) and psychology students (a sample likely to be more familiar with inattentional blindness) also completed the survey. All participants were asked scenario-based questions to assess beliefs about visual detection in legal settings in which inattentional blindness might occur. Following this, participants were asked questions regarding their familiarity with inattentional blindness, personal experiences with inattentional blindness, and beliefs as to whether individuals can do anything to enhance noticing of unexpected events. These latter questions were included to identify gaps in knowledge that could be a focal point of professional training/education, and to ascertain relatable and common experiences of inattentional blindness that could be drawn on in legal practice. Altogether, this exploratory survey advances our knowledge of beliefs about inattentional blindness in legal contexts, by providing a preliminary understanding of the beliefs held by important legal populations. Given the exploratory and largely descriptive nature of this work, we did not pre-register any specific hypotheses (see https://osf.io/gvpd6).

## Method

### Participants

Survey participants were drawn from four different populations: police officers (*n* = 94), lawyers (*n* = 98), psychology students (*n* = 99), and members of the community (*n* = 100). Given that the survey largely consists of frequency data, and that our legal populations are typically hard to reach, we decided a-priori to collect up to 100 participants per population. Power calculations conducted using G*Power 3.1 [28] revealed that 100 participants per population was sufficient for conducting ANOVA and chi-square analyses when assuming a power of .80 and medium effect sizes. Table 1 displays the key demographic characteristics for each of the four samples.

**Table 1:** Demographic characteristics of police, lawyer, psychology, and community participants.

	**Police**		**Lawyer**		**Psychology**		**Community**	
	M	SD	M	SD	M	SD	M	SD
Age	44.85	8.99	32.38	7.51	20.42	5.19	28.85	9.48
Gender	n	%	n	%	n	%	n	%
Male	64	68.1	45	45.9	28	28.3	55	55.0
Female	30	31.9	53	54.1	71	71.7	43	43.0
Non-binary	-	-	-	-	-	-	2	2.0
Ethnicity	n	%	n	%	n	%	n	%
Aboriginal or Torres Strait Islander	2	2.1	-	-	-	-	-	-
East Asian	-	-	5	5.1	13	13.1	8	8.0
European/White	86	91.5	83	84.7	55	55.6	72	72.0
Hispanic	-	-	-	-	2	2.0	-	-
Middle Eastern	-	-	2	2.0	7	7.1	4	4.0
Mixed	3	3.2	4	4.1	13	13.1	3	3.0
Pacific Islander	-	-	1	1.0	1	1.0	-	-
South Asian	2	2.1	-	-	2	2.0	7	7.0
Southeast Asian	1	1.1	3	3.1	6	6.1	6	6.0
Total	94		98		99		100	

### Police officers

Police officers employed by the Australian Federal Police were recruited voluntarily via an advertisement posted on their staff intranet. Overall, 141 police officers began the survey, but 47 participants did not complete the survey, resulting in a final sample of 94 police officers. This sample size is similar to previous surveys involving Australian police officers [29–31]. Our police sample was comparable in terms of age and gender to Australian police officers as per recent census data [32]. Specifically, most police officers were male (68.1%), with an average age of 44.85 years (*SD* = 8.99). Police officers had an average of 18.26 years’ experience as a police officer (*SD* = 10.42, *range*: 1–43). Most police officers were based in the Australian Capital Territory (55.3%), as the Australian Federal Police mostly operates out of this state. However, other officers were also based in New South Wales (16%), Queensland (10.6%), Western Australia (7.4%), Victoria (5.3%), and other states/territories (4.3%; 1.1% prefer not to say). Common roles among the police officers recruited were investigations (29.8%), detective (12.8%), general duties (10.6%), child protection (4.3%), and community policing (4.3%) (other categories: 34.0%, prefer not to say: 4.3%). No differences were observed in demographic or work history factors between police officers who completed the survey in full and those who did not (all *p*s > .108).

### Lawyers

Au**s**tralian lawyers were recruited for the survey via social media (Facebook, LinkedIn, Reddit, & Twitter). Overall, 164 lawyers began the survey, but 65 participants failed to complete the survey. In addition, one further participant provided invalid responses to the survey. Together, this left a valid sample of 98 lawyers. This sample size is similar to previous surveys of lawyers in Australia [27, 33]. Of these 98 lawyers, 53 were female (54.1%; 45.9% male), and the Mean age was 32.38 years (*SD* = 7.51). The gender breakdown is similar to that of recent Australian census data, but our sample was slightly younger than the broader Australian population of solicitors [34]. Lawyers who completed the survey were predominantly European/White (84.7%).

Lawyers were required to be practicing law within an Australian State or Territory and fluent in English to participate. We chose not to place any restrictions on experience or practice areas of our legal sample, because it is possible that cases involving inattentional blindness may come up in many different types of law. We also wanted to gain insight into the breadth of experiences and beliefs within the lawyer sample. Lawyers had practiced law from 1 to 31 years, with a Mean practice time of 6.24 years (*SD* = 5.84). Lawyers were predominantly solicitors (88.8%), and most practiced in New South Wales (53.1%). There was variability in the area(s) of law in which they practiced, with the most common areas being Civil Litigation (32.7%), Criminal Law (26.5%) and Family Law (21.4%).

There were no differences between lawyers who did and did not complete the survey (all *p*s > .105), except for state of practice, *Fisher’s Exact Test =* 17.530, *p* = .006, *φ*_*c*_ = .365_._ Specifically, the standardised residuals indicated that there were more “prefer not to say” responses to the question about state of practice among non-completers, compared to completers.

### Psychology students

Introductory psychology students were recruited via the University of Sydney research participation pool. These students were recruited for two reasons. First, as inattentional blindness is a psychological phenomenon, this population may have increased knowledge about it due to their studies. Second, psychology students have been the focus of previous research on beliefs about inattentional blindness. Therefore, including psychology students allowed us to determine whether beliefs about inattentional blindness from previous research are generalisable and comparable across samples. Only Australian residents who were fluent in English were eligible to participate in the study. Overall, 100 psychology students began the survey, but one participant was excluded from analyses for failing to complete the survey. Psychology students were predominantly female (71.7%, 28.3% male), with a Mean age of 20.42 years (*SD* = 5.19). The gender and age breakdown of our psychology students was similar to that in previous research [12, 13, 18]. Psychology students were most commonly European/White (55.6%).

### Community members

Members of the community were recruited via Prolific and were compensated £1.25 for their participation. Only Australian residents who were fluent in English were eligible to participate, to be consistent with the main juror eligibility criteria. In total, 100 community participants (55% male; 43% female; 2% non-binary) completed the survey. This sample size of community participants is similar to that of other recent surveys on lay perceptions regarding memory [35, 36]. They had a Mean age of 28.85 years (*SD* = 9.48), were predominantly European/White (72%), and were mostly well educated (55% had received a Bachelor’s degree or above).

The community sample was similar to the broader Australian population with regard to gender, based on previous Australian census data [37]. However, educational attainment in our sample appeared to be greater than the broader Australian population, as only 36% of the broader Australian population has received a Bachelor’s degree or above [38]. It is common for participants recruited through Prolific to be highly educated, and our educational attainment data is similar to another study recruiting members of the United States public through Prolific [39]. Our community sample was also younger than the broader Australian population [37]. We therefore label this group as a community sample as opposed to a general population sample, so as not to generalise the beliefs held by this sample to those held by the broader Australian population or jury pool.

### Survey and procedure

Participants volunteered to take part in a survey titled “*What do people notice*, *and when*?*”*. All participants began by providing their age, gender, and cultural background. Lawyers and police officers provided additional information about their work experience. Members of the community were asked to provide their educational background, as they were the only population without clear qualifications and who were not recruited based on a specific area of study or employment. Psychology students were not asked these questions related to education/work.

Next, all participants were asked to read six scenarios and state whether they believed the individual in the scenario would notice the unexpected event in question (yes or no). Each scenario was presented in a randomised order and was based on a published study on inattentional blindness, so that we could get a sense of how beliefs about noticing the unexpected event align with the published literature. Additionally, each scenario was chosen due to its legal relevance. All scenarios involved a situation in which failing to notice the object or event could have legal repercussions, such as a witness failing to notice a criminal event, or an individual being sued or charged with negligence. A description of the scenarios and studies that they were based on is presented in Table 2.

**Table 2:** Scenarios and studies of inattentional blindness they are based on.

Scenario Label	Full scenario	Inattentional blindness studies	% of participants in original study who noticed event	Reference group in original study
CCTV	A CCTV operator is watching CCTV footage to identify security threats. An individual drops off a package while the operator views the footage.	[40]	39%	Experienced observers, relevant stimulus, combined clip length
Radiologist	A foreign object is visible in a patient’s medical scan. A radiologist checks over the scan for nodules (growths of abnormal tissue).	Combination of [41, 42]	[41]: 21.9% [42]: 16.7%	[41]: Foreign body noticed across both procedure groups (standard endoscopy and augmented reality) [42]: Study 1 –all participants
Witness	An individual is running, chasing after someone in a park. An assault occurs in the park near the runner.	[4]	45.5%	Study 2 –all participants
Driver	A driver in a car is determining whether the road is safe to drive on. A lady with a stroller is at the traffic lights on the median strip (the narrow sidewalk between two roads).	[5]	68%	Non-expert, stroller (medium-high threat)
Pilot	A pilot is flying a plane using heads-up navigational display (a screen that projects flight information at eye-level). The aircraft starts to rapidly descend.	[43]	58.3%	Pilots who noticed and took action before collision with the ground
Police	A police officer has stopped a car for failing to stop at a stop sign. A gun is on the dashboard of the car that the police officer stops.	[44]	52.6%	All police (officers and trainees), combined driver behaviour (compliant and aggressive)

*Note*: All scenarios are presented in a randomised order.

When assessing the beliefs about noticing the unexpected event in each scenario, participants could only provide a “yes” or “no” response. We chose to have participants answer in yes/no format for two key reasons. First, we wanted the beliefs we were capturing to map onto the kinds of decisions that would be made in real life. More specifically, in real life cases where an individual’s visual experience is in question, legal professionals and jurors will need to make concrete, binary decisions such as whether they believe a person acted negligently or not based on their visual experience [17], or whether they believe someone’s visual experience reflects that of a “reasonable person” or not [45]. Thus, yes/no judgments capture the type of real-world decision-making that the samples of interest in this paper (police officers and lawyers) would be engaged in, as well as the binary nature of the decisions that would be reached by other populations relevant to this research (e.g., jurors). Second, previous studies of visual metacognition have used a yes/no response format for assessing beliefs about noticing [12, 15, 46].

It should be noted that in striking a balance between making the scenarios realistic and keeping them true to the actual studies of inattentional blindness, it was not possible to do this for all scenarios. For example, the medical scenario was based upon two separate studies of inattentional blindness [41, 42], because the details were overly technical in one study [41], and the unexpected event was implausible for another (a gorilla in a chest X-ray) [42]. Thus, the unexpected event (a foreign object) and task (looking for abnormalities in scans) were combined from these studies to create the medical scenario for the current survey. Additionally, the pilot and driver scenarios in our study refer to a pilot and driver operating an actual plane and car, respectively, while in the original studies, the pilots used a flight simulator [43] and the drivers made decisions about the safety of images of driving scenes [5]. Altogether, this means that while the rates of noticing the unexpected events are informative, the lack of realism in some of these original studies means that we cannot directly compare the rates of noticing to the beliefs about noticing in this survey.

Participants were then asked nine questions to determine their beliefs about whether certain factors would make individuals more likely, just as likely, or less likely to notice an unexpected event/object. The order in which these questions were presented was randomised and the factors were based on previous studies on inattentional blindness. For some factors, the existing research would suggest that individuals would be more likely to notice the unexpected event, while for others, the individuals would be less likely to notice the unexpected event. Additionally, for some factors, findings within the research have been mixed, or there is a lack of research. The response options to choose from for each factor were “more likely”, “just as likely” and “less likely”. See Table 3 for a full list of scenarios and the consensus within the research.

**Table 3:** Factors influencing inattentional blindness and support from research studies.

Factors	Research support
Event closer to focal point vs. further away (Distance)	More likely to notice [47, 48]
Event more meaningful to person vs. less meaningful (Meaning)	More likely to notice [1]
Event in lighter conditions vs. darker conditions (Lighting)	More likely to notice [4]
Individual consumed alcohol vs. sober (Intoxication)	Less likely to notice [49, 50]
Hard vs. easy task (Task Difficulty)	Less likely to notice [2, 51]
Event more threatening to person vs. less threatening (Threat)	Mixed findings [52, 53]
Individual experienced in task vs. less experienced individual (Experience)	Mixed findings [54, 55]
Individual with better memory vs. poorer memory (i.e., WMC) (Memory)	Mixed findings [56–60]
Individual very intelligent vs. less intelligent (Intelligence)	No research (except one study with older adults) [61]

*Note*: Brackets next to factors indicate labels given to each factor in the subsequent analyses.

Next, participants were asked whether they were familiar with the term “inattentional blindness” (yes or no). Participants who indicated that they were familiar with the term were asked to provide a definition of the term, to state how they learnt about the term, and to describe any studies of inattentional blindness, and any day-to-day activities during which inattentional blindness may occur. These were all free-response questions. Participants who were not familiar with the term were asked to provide a free-response definition of what they thought inattentional blindness could mean. All participants were asked whether they were familiar with the famous gorilla experiment of inattentional blindness (yes or no) [2]. Participants who reported being familiar with the gorilla experiment were asked to report what they knew about the experiment in a free-response format.

All participants were then provided with the following definition of inattentional blindness: *“Inattentional blindness refers to when people fail to notice unexpected events that happen right in front of them because they are paying attention to something else”*. Based on this definition, participants were asked if they believed inattentional blindness is common in everyday life (yes or no), and whether they, themselves, had experienced inattentional blindness before (yes or no). Those who responded that they had experienced inattentional blindness before were asked whether they could think of an example of a time when they had experienced it (yes or no). If they could think of a time when they had experienced inattentional blindness, participants were asked a series of free-response questions concerning the most serious or noteworthy experience they could think of (what they failed to notice, what were they focusing on when it happened, if there were any negative consequences resulting from failing to notice the event [yes or no], and if so, what these consequences were). Participants who responded that they had never experienced inattentional blindness before were asked why they believed they had never experienced it (in free-response format).

Finally, participants were asked whether they believed there was anything that a person could do to make themselves more likely to notice something they were not expecting (yes or no). If participants responded that they believed something could be done, they were asked exactly what they believed could be done. All participants were given a final opportunity to report anything else about the topic of the survey before submitting their questionnaire.

The survey was pre-registered, and all materials can be found at https://osf.io/vh3ac/. All aspects of the survey were approved by The University of Sydney Human Research Ethics Committee (protocol number: 2020/114). Written consent (via electronically indicating consent in the checkbox provided) was obtained from all participants. Data collection took place from 20 March 2020 to 18 February 2021.

## Results

### Demographics

Preliminary analyses were conducted to determine whether there were differences in demographic characteristics (e.g., age, gender, cultural background) between each population. There were significant demographic differences across each of the populations. First, there was a significant difference in age across the populations, *F*(3,387) = 156.207, *p* < .001, *η*_*p*_^*2*^ = .548, BF_10_ > 100, such that police officers were significantly older than lawyers (*p* < .001, BF_10_ > 100), who were significantly older than community members (*p* = .010, BF_10_ = 7.470), who were significantly older than psychology students (*p* < .001, BF_10_ > 100) (see Table 1 for demographic information). Second, there was a significant relation between gender and population, Fisher’s Exact Test = 37.047, *p* < .001, *φ*_*c*_ = .223, BF_10_ = 37.867. The standardised residuals indicated that the psychology sample contained more females and fewer males than expected, while the police sample contained more males and fewer females than expected (though noting that these samples are typical of the psychology and policing professions) [32, 62]. Third, there was a significant relation between cultural background and population, χ^2^ (3, *N* = 391) = 37.696, *p* < .001, *φ*_*c*_ = .310, BF_10_ > 100. The standardised residuals indicated that the psychology sample contained more non-White participants than expected, while the lawyer and police samples contained fewer non-White participants than expected.

### Beliefs about noticing unexpected events

For six different legal scenarios, participants were asked to state whether the individual in the scenario would have noticed the unexpected event in question (yes or no). The proportion of participants from each legal population believing the individual would have noticed the event is displayed in Table 4. It is not appropriate to directly compare these beliefs about noticing to the actual noticing rates in each of the studies as presented in Table 2, because participants in our survey were only asked about a single individual’s visual experience and the rates of noticing in the original studies are percentages of the overall rate across participants. However, the frequency data in Table 4 indicate that across all populations, there is a tendency to believe that the individual in each scenario would have noticed the event in question (as opposed to fail to notice). This was true for all but the *witness* scenario, in which beliefs about noticing were lower, especially among the police (40.4%) and lawyer (34.7%) populations.

**Table 4:** Respondents (%) from each population believing that the individual would have noticed the unexpected event (providing a “yes” response).

	Population			
Scenario	Police	Lawyer	Psychology	Community
CCTV	80.9	71.4	80.8	84.0
Radiologist	95.7	94.9	96.0	95.0
Witness	40.4	34.7	56.6	52.0
Driver	69.1	85.7	77.8	83.0
Pilot	97.9	94.9	87.9	89.0
Police	89.4	91.8	85.9	89.0

Lawyers working in some fields (e.g., torts, negligence, or criminal law) may come across cases involving inattentional blindness more frequently than lawyers in other fields. To account for this, we conducted exploratory analyses to compare civil and criminal lawyers to lawyers working in any other area of law, on their beliefs about visual detection for each of the six legal scenarios. Fisher’s Exact Tests revealed no significant relation between area of law and beliefs (all *p*s > .061, two-sided). For the Bayesian analyses, only for the police scenario was there anecdotal evidence for a difference between civil/criminal lawyers and other lawyers (BF_10_ = 1.168), with those in civil/criminal law less likely to believe the police officer would have noticed the gun (86%) than the other lawyers (98%). For the other scenarios, Bayes Factors either revealed ambiguous evidence, or moderate evidence for a lack of difference. Therefore, for the most part, it does not appear that beliefs about noticing legally relevant events differ for lawyers based on area of legal practice.

### Beliefs about factors that may affect noticing of unexpected events

Participants responded to nine questions regarding whether they believed certain factors would make an individual more likely, just as likely, or less likely to notice unexpected events. Responses to each of these questions for each population are presented in Table 5. Most respondents in each population appeared to endorse the most appropriate option for the questions, except for the level of threat of the object, and the level of expertise of the observer. While some studies do indicate that these factors *can* make an individual more likely to notice the unexpected event, there are also findings to the contrary.

**Table 5:** Responses based on factors influencing inattentional blindness.

	Population											
	Police			Lawyer			Psychology			Community		
Factors	More	Just as	Less	More	Just as	Less	More	Just as	Less	More	Just as	Less
Best answer: More likely												
Distance	83.0	14.9	2.1	84.7	14.3	1.0	92.9	5.1	2.0	85.0	11.0	4.0
Meaning	91.5	6.4	2.1	82.7	16.3	1.0	80.8	17.2	2.0	74.0	23.0	3.0
Lighting	72.3	20.2	7.4	63.3	27.6	9.2	76.8	14.1	9.1	80.0	15.0	5.0
Best answer: Less likely												
Intoxication	-	2.1	97.9	1.0	8.2	90.8	2.0	6.1	91.9	1.0	3.0	96.0
Difficulty	8.5	20.2	71.3	11.2	14.3	74.5	10.1	7.1	82.8	5.0	13.0	82.0
Best answer: Just as likely ^a^												
Threat	87.2	8.5	4.3	85.7	11.2	3.1	89.9	7.1	3.0	78.0	17.0	5.0
Experience	63.8	28.7	7.4	64.3	24.5	11.2	65.7	16.2	18.2	73.0	16.0	11.0
Memory	14.9	84.0	1.1	15.3	84.7	-	18.2	81.8	-	18.0	80.0	2.0
Intelligence	11.7	85.1	3.2	23.5	74.5	2.0	17.2	81.8	1.0	31.0	68.0	1.0

^a^For threat and experience, there are mixed findings regarding how they affect noticing. Therefore, while we acknowledge that not all research suggests that these factors would fail to influence noticing, for the purpose of this survey and the response options provided to participants, “just as likely” was the most suitable response option to account for these mixed findings.

### Knowledge of inattentional blindness

Participants were asked whether they had heard of the term inattentional blindness before. Across all populations, 35% of participants reported knowing the term, while 65% reported not knowing with the term. A chi-square analysis exploring differences in knowledge of inattentional blindness (knew term vs. did not know term) across populations revealed no significant relation, *χ*^2^ (*N* = 391) = 5.960, *p* = .114, *φ*_*c*_ = .123. Additionally, there was moderate evidence favouring no difference between the populations, BF_01_ = 7.729. Therefore, self-reported knowledge of the phenomenon did not significantly differ across the populations.

All participants who knew the term inattentional blindness were asked to provide a definition of inattentional blindness. Likewise, participants who did not know the term were asked to provide a definition of what they believed inattentional blindness might mean. To determine the extent of participants’ knowledge regarding the phenomenon, responses to these questions were coded. The coding was based on Simons (2007), who suggested that there are four key criteria for inattentional blindness [63]:

1. an individual *fails to notice* an event,
2. the event is *unexpected*,
3. the event occurs in *plain sight*, and
4. the individual fails to notice because their *attention is elsewhere*.

Definitions were coded based on how many of the four criteria for inattentional blindness participants reported, as well as which aspects of the four criteria participants reported. Using McHugh’s (2012) framework for interpreting Cohen’s kappa [64], two independent coders (HC & TD) each scored the number of criteria reported by respondents, as well as which criteria were reported. The two coders reached moderate agreement and above on the number and aspects of the criteria participants reported (Cohen’s κ = .737, *N* = 391, *p* < .001).

Across all participants (regardless of whether they reported that they knew what inattentional blindness meant), most possessed some level of understanding of the concept of inattentional blindness. Specifically, 6.4% of participants could not provide any of the four criteria for inattentional blindness, 13.6% provided one criterion, 62.7% provided two criteria, and 15.6% provided three criteria. Notably, only 7 participants across all samples (1.8%) provided a completely correct definition that contained all four criteria. When looking at the aspects of the criteria that were commonly reported within participant definitions, 90.5% of all respondents said *failure to notice*, 75.7% said *attention elsewhere*, 19.4% said *plain sight*, and 10% said *unexpected*. Table 6 reports self-reported familiarity, number of criteria correct, and the frequency of the criteria reported across each of the four populations. A one-way ANOVA revealed a significant effect of population on number of criteria reported, *F*(3,387) = 3.401, *p* = .018, *η*_*p*_^*2*^ = .026, although the Bayes factor revealed anecdotal evidence favouring no difference, BF_01_ = 1.025. Bonferroni corrected post-hoc contrasts revealed that psychology students were able to provide more criteria for inattentional blindness than community members (BF_10_ = 7.204) and police officers (BF_10_ = 4.839), with all *p*s < .044, but did not report significantly more criteria than lawyers, *F*(1,387) = 3.350, *p* = .068, *η*_*p*_^*2*^ = .009, BF_01_ = 1.063. There were no differences in number of criteria correctly reported between lawyers and police officers (*p* = .379, BF_01_ = 4.508), between community members and lawyers (*p* = .326, BF_01_ = 4.163), or between community members and police (*p* = .930, BF_01_ = 6.386).

**Table 6:** Familiarity and understanding of inattentional blindness.

	Self-reported familiarity (%)	Number of criteria correct		Criteria reported (%)			
Population		*M*	*SD*	Failure to notice	Unexpected	Plain sight	Attention elsewhere
Police	34.0	1.83	0.84	87.2	10.6	22.3	61.7
Lawyer	34.7	1.93	0.75	90.8	12.2	16.3	77.6
Psychology	43.4	2.13	0.69	97.0	10.1	22.2	87.9
Community	27.0	1.82	0.82	87.0	7.0	17.0	75.0

Participants were also asked questions about whether they knew any studies of inattentional blindness, and specifically the gorilla experiment [2]. Regardless of whether they knew the term inattentional blindness, 34% of all participants reported familiarity with the gorilla experiment. A chi-square revealed no significant relationship between population and knowledge of the gorilla experiment of inattentional blindness, *χ*^2^ (3, *N* = 391) = 4.982, *p* = .173, *φ*_*c*_ = .113. This was corroborated by strong evidence in favour of no relationship, BF_01_ = 11.849. Very few other studies of inattentional blindness were mentioned in participants’ responses, but some studies included the static inattentional blindness experiment of Mack and Rock (1998) [1], and change blindness experiments [65].

Next, participants who reported knowing about inattentional blindness were asked how they had learned about the phenomenon. As responses were provided in free-text format, one coder (HC) went through all responses to generate eight separate experiences (study, real-world experiences, videos/TV/movies, reading, social media/internet, general knowledge, discussions with others, unsure). Two coders (HC & TD) then independently applied these eight experiences to all responses, and inter-rater reliability ranged from moderate to almost perfect (all Cohen’s κs > .60, all *p*s < .001). Table 7 outlines the proportion of participants who reported knowing what inattentional blindness meant based on each of the above experiences. Overall, knowledge about inattentional blindness was most commonly derived from study, followed by real-world experiences, and consumption of information through various sources (e.g., video, reading, social media). However, contrary to the other populations, for police officers, knowledge of inattentional blindness from real-world experiences (e.g., work and training courses) outweighed knowledge of inattentional blindness through study. Lawyers were also equally likely to say that they were unsure of how they learned about inattentional blindness as they were to say that they learned about it through study.

**Table 7:** Experiences where familiarity with inattentional blindness derived from (% of respondents).

	Population				
Experience	Police (*n* = 32)	Lawyer (*n* = 34)	Psychology (*n* = 43)	Community (*n* = 27)	Total
Study	18.8	29.4	79.1	40.7	44.9
Real-world experiences	46.9	20.6	4.7	11.1	19.9
Videos, TV, or movies	9.4	11.8	9.3	29.6	14.0
Reading	15.6	5.9	4.7	14.8	9.6
Social media/internet	3.1	8.8	4.7	11.1	6.6
General knowledge	6.3	2.9	2.3	3.7	3.7
Discussions	6.3	2.9	2.3	3.7	3.7
Unsure	6.3	29.4	9.3	3.7	12.5

### Anecdotal experiences of inattentional blindness

Overall, the vast majority (97.4%) of respondents reported that they believed experiencing inattentional blindness was common in everyday life, and most respondents (93.6%) stated that they had experienced inattentional blindness in the past. However, only about half of all respondents (50.5%) were able to provide a specific example of a time that they had experienced inattentional blindness. A chi-square analysis revealed no significant relation between population and ability to provide an example of inattentional blindness in their personal lives, *χ*^2^ (3, *N* = 366) = 4.623, *p* = .202, *φ*_*c*_ = .112. This was further evidenced by strong evidence for no difference, BF_01_ = 15.529.

Anecdotal experiences of inattentional blindness (i.e., participants’ own personal experiences of inattentional blindness, as opposed to their beliefs in the previous section) were coded according to the task participants were engaged in, and the critical event that they failed to notice. A similar coding process as above was used: one coder (HC) identified broad task and event categories based off the free-text responses provided. Two independent coders (HC & TD) applied these task categories to the task responses and the event categories to the event responses. Task and event categories that were infrequently reported (i.e., by less than 5% of participants) are not reported below. However, seven common task categories were identified: driving, working/studying, using technology, reading, completing housework, thinking/concentrating, and engaging in social activities. Additionally, six event categories were identified: driving-related hazard, general hazard, crime, social event, housework-related events, and work/study related event. The inter-rater agreement for each category was moderate to high (Cohen’s κs > .60, all *p*s < .05), for all but two categories (general hazard and housework-related events), which were weak in agreement (κ = .49, *p* < .05; κ = .55, *p* < .05). The frequencies of each personal experience of inattentional blindness are presented in Table 8. The most common two tasks respondents reported completing during personal experiences of inattentional blindness were work/study and driving, and the most common two events that were missed (i.e., not noticed) were driving hazards (e.g., street signs, pedestrians) and social events (e.g., a friend trying to get their attention). These examples may therefore be the most relatable examples of inattentional blindness, as they are commonly reported.

**Table 8:** Personal experiences of inattentional blindness (% of respondents who could describe personal experience).

	Population				
Category	Police (n = 44)	Lawyers (n = 40)	Psychology (n = 49)	Community (n = 52)	Total
Task					
Working/studying	36.4	32.5	18.4	21.2	26.5
Driving	20.5	20.5	34.7	17.3	23.4
Engaging in social activities	15.9	17.5	18.4	9.6	15.1
Thinking/concentrating	15.9	15.0	12.2	13.5	14.1
Using technology	9.1	10.0	8.2	15.4	10.8
Completing housework	11.4	2.5	2.0	9.6	6.5
Reading	2.3	10.0	6.1	5.8	5.9
Event					
Driving-related hazard	25.0	22.5	38.8	30.8	29.7
Social event	22.7	22.5	16.3	25.0	21.6
Work/study related event	18.2	17.5	10.2	13.5	14.6
Housework-related event	2.3	10.0	12.2	13.5	9.7
Crime	13.6	7.5	4.1	7.7	8.1
General hazard	11.4	5.0	6.1	3.8	6.5

Participants who provided an example of inattentional blindness were asked whether there were any negative consequences resulting from their experience. Of those who provided an example (50.5%), 30.3% (*n* = 56) stated that there were negative consequences of their experience. Examination of the qualitative responses indicated that serious consequences included being involved in car accidents (23.2%), being involved in an incident at work (12.5%), becoming a victim of crime (12.5%), or becoming injured (10.7%), among others. Therefore, when individuals can draw on their personal experiences of inattentional blindness, these experiences are not always benign, and can in fact be quite serious.

### Beliefs about enhancing noticing of unexpected events

Participants were asked whether they believed that individuals could do anything to make themselves more likely to notice an unexpected event (and if so, what). Interestingly, 58.8% of all participants (*n* = 230) believed that people could do something to make themselves more likely to notice unexpected events. A chi-square revealed a significant relation between population and belief that noticing is within a person’s control, *χ*^2^ (3, *N* = 391) = 14.253, *p* = .003, *φ*_*c*_ = .191, further reinforced with moderate evidence for a relationship, BF_10_ = 8.424. The standardised residuals indicated that psychology students responded “no” (i.e., that people cannot do anything to enhance noticing of unexpected events) above expected counts, while police officers responded “yes” (i.e., that people *can* do something to enhance noticing of unexpected events) above expected counts. No significant relation was observed between self-reported familiarity with inattentional blindness and belief that individuals can do something to enhance noticing of unexpected events, *χ*^2^ (1, *N* = 391) = .047, *p* = .829, *φ*_*c*_ = .011, which is supported by the Bayes factor indicating moderate evidence for no relationship, BF_01_ = 7.517.

In coding the free-text data regarding what strategies individuals could employ to enhance noticing of unexpected events, one coder (HC) identified several general categories that emerged in participant responses. Two independent coders (HC & TD) applied these categories to all responses. Strategies that were infrequently reported (i.e., by less than 5% of participants) are not reported below. However, seven more common strategies emerged: being aware of surroundings, training, avoiding focused attention, avoiding distractions/multitasking, meditation/mindfulness, taking breaks, and learning about inattentional blindness. Inter-rater agreement for each category was moderate to high (Cohen’s κs > .70, all *p*s < .05). Responses regarding each strategy for enhancing noticing of unexpected events across each population are presented in Table 9. For all populations, the most commonly endorsed strategy involved having a heightened awareness of one’s surroundings. However, the subsequent most commonly endorsed strategies appeared to differ among the populations. Legal populations (police officers and lawyers) endorsed training as the second most common strategy. Lawyers also commonly listed meditation and mindfulness techniques as a strategy. Interestingly, while the second most common strategy for psychology students was to avoid multitasking or sources of distraction, in stark contrast, the second most common strategy stated by community members was avoiding focusing too much on what one is doing.

**Table 9:** Respondents by population endorsing each option for enhancing noticing of unexpected events (%).

	Population				
Response	Police (n = 69)	Lawyer (n = 59)	Psychology (n = 48)	Community (n = 53)	Total
Be aware of surroundings	46.4	35.6	50.0	49.1	45.0
Training	23.2	16.9	8.3	3.8	14.0
Avoiding focused attention	13.0	6.8	8.3	15.1	10.9
Avoiding distractions/multitasking	2.9	8.5	20.8	11.3	10.0
Meditation/mindfulness	2.9	16.9	2.1	9.4	7.9
Taking breaks	8.7	6.8	4.2	7.5	7.0
Learning about inattentional blindness	5.8	6.8	6.3	7.5	6.6

## Discussion

It is common for people to fail to notice unexpected events occurring right in front of them when focusing their attention elsewhere. However, existing research on beliefs about inattentional blindness, as well as related visual metacognitive research on change blindness blindness, has suggested that our visual experiences are poorly understood, leading us to overestimate the likelihood that we will notice unexpected objects and changes in our environment [10–12, 14, 15]. Given that occurrences of inattentional blindness can have serious legal consequences [17, 22], it is important to gain a preliminary understanding of the beliefs regarding inattentional blindness and visual detection held by legal populations who may be involved in cases of this nature. It is also important to canvas the current knowledge base and familiarity with inattentional blindness to identify opportunities for training and education in legal contexts. To achieve these goals, we surveyed police officers and lawyers, who have been so far unexplored in the area of visual metacognition. We also included psychology students and members of the community.

### Summary of findings

First, the current survey revealed that it was common for respondents across all four populations to believe that individuals would notice unexpected visual events that are legally relevant. That is, for almost all scenarios, most police officers, lawyers, psychology students, and community members sampled stated that they believed the individual in the scenario would have noticed the unexpected event in question. While we cannot make direct comparisons back to the actual studies on inattentional blindness, when looking to the rates of noticing the unexpected events in the original experiments (see Table 2), participants in our survey appear to be optimistic about the likelihood that unexpected events will be noticed in legally relevant scenarios. This was true for all but one scenario: the *witness* scenario. Specifically, 46% of our participants reported that the witness would have seen the crime, while 45.5% of participants in the original study noticed the crime in the same scenario [4]. Police officers and lawyers—the two legal populations—also had the lowest beliefs in visual detection; just 40.4% and 34.7% of police officers and lawyers, respectively, stated that they believed the witness would have noticed the crime. Interestingly, lawyers in areas of practice where they would likely encounter more cases involving inattentional blindness (e.g., criminal and civil law) did not appear to hold different beliefs to lawyers in other areas of specialisation for most of the scenarios.

Most respondents had a good understanding of how certain factors may affect one’s ability to notice an unexpected event. For most factors, most participants across all four populations endorsed the response option that is best supported by the existing empirical literature. Specifically, most participants believed that a closer and more meaningful unexpected event would be more likely to be noticed; that alcohol intoxication and a hard task would make an unexpected event less likely to be noticed; and that memory and intelligence would not affect the likelihood of noticing an unexpected event. While participants responded in line with the literature for most factors that influence inattentional blindness, this was not the case for two factors: observer experience/expertise and threat.

With respect to knowledge of inattentional blindness, 35% of participants reported that they were familiar with the term, and 34% were familiar with a previous study of inattentional blindness (often the now famous gorilla experiment of inattentional blindness by Simons & Chabris) [2]. While there were no differences across the populations with respect to self-reported familiarity with inattentional blindness, psychology students provided more of the criteria for inattentional blindness in their definitions. Additionally, while learning about inattentional blindness through education/study was the most common reason for familiarity with the concept, this was most prominent among psychology students. Police officers instead commonly reported learning about inattentional blindness through real-world experiences (such as work and training). While respondents across all populations were fairly good at indicating that inattentional blindness would relate to failing to notice an event due to a lack of attention (two of four specific criteria for inattentional blindness), the components of the event being *unexpected* and in *plain sight* were often neglected from the definitions that respondents generated. This is concerning given that these two criteria are the least likely to be inferred from the term itself and may be particularly counterintuitive.

Participants were also asked whether they could provide an example of a time that they had experienced inattentional blindness. We included this question to determine whether there are common and relatable examples of inattentional blindness that might be best drawn upon in training and education on the topic. The findings from our survey indicate that people may have trouble recalling their own experiences of inattentional blindness. Only 50.5% of our participants could recall a personal experience of inattentional blindness, despite almost all participants (93.6%) indicating that they have experienced it in the past. Closer inspection of the examples that participants could bring to mind revealed some common themes, such as failing to notice hazards while driving or while on the phone. Importantly, some participants reported experiencing very salient and serious instances of inattentional blindness, with severe consequences (e.g., crime, fire, and injury). Therefore, when individuals can reflect on their own experiences of inattentional blindness, these experiences are not always trivial in nature.

Finally, participants were asked whether they believe that individuals can do anything to enhance their ability to notice unexpected events. While there is no empirical evidence to suggest that inattentional blindness can be prevented [66], just over half (58.8%) of all respondents within the survey reported that they believed that there are ways to make individuals more likely to notice unexpected events.

### Implications

Theoretically, the findings of the current survey are important in highlighting that there may be some misconceptions and mistaken beliefs about inattentional blindness and visual detection held by legal populations. While the existing research on beliefs about inattentional blindness has focused on undergraduate or community samples [10–13], our findings suggest that legal populations, too, hold misconceptions and have gaps in their knowledge, reinforcing that inattentional blindness is a counterintuitive and surprising phenomenon [7]. Previous mock juror research has revealed that claims of inattentional blindness made in legal contexts are evaluated negatively, both in criminal and civil cases. Specifically, individuals who claim that they failed to notice a physical assault/fight in their visual field are perceived as less credible witnesses in criminal trials [16] and more negligent defendants in civil cases [17]. Thus, the mistaken beliefs and misconceptions that people have about inattentional blindness may have flow-on effects to legal decision-making. Police officers and lawyers hold misconceptions and mistaken beliefs about inattentional blindness and visual detection that could similarly compromise their decision-making in legal cases. As our findings are a starting point in understanding beliefs about inattentional blindness in legal contexts, it would be pertinent for future research to build on our findings and evaluate actual decision-making and behaviour of these legal professionals in cases of potential inattentional blindness.

The misconceptions and gaps in knowledge/understanding around inattentional blindness and visual detection that we have identified in our survey provide critical insight into what education and training on the topic should focus on. First, it was common for legal professionals to believe that expertise/experience and threat would both increase the likelihood of noticing unexpected events. Such beliefs are not conclusively backed up by research, as empirical findings are inconsistent [52–55]. Indeed, legal cases of potential inattentional blindness may involve people with many years of experience or expertise in the task they are completing (e.g., drivers or medical professionals) or consist of threatening events (e.g., crimes and weapons). Thus, it will be important to educate legal professionals that the presence of these factors does not guarantee that individuals will notice unexpected events in the absence of attention.

Additionally, most legal professionals did not report all the criteria for inattentional blindness, particularly that it involves *unexpected* events, and events that occur in *direct view*. Legal training and education should emphasise these less commonly reported components of inattentional blindness, potentially through drawing on the most relatable examples of inattentional blindness identified in our survey (e.g., in work, driving, and social contexts). This is important, given these two criteria are more counterintuitive, but can be especially relevant in legal cases. Such education could also feature in expert witness testimony in legal cases of potential inattentional blindness; though the limited empirical research conducted thus far suggests that expert testimony may not sensitise mock jurors to claims of inattentional blindness [16].

Finally, as respondents also reported strategies for improving noticing of unexpected events that empirical research suggests will not be effective (e.g., being familiar with the concept of inattentional blindness) [18, 21, 67], training and education programs should remind legal professionals of the difficulty in preventing inattentional blindness. Alternatively, since it is unclear whether inattentional blindness can be prevented at the level of the individual, there may be ways that organisations could reduce rates of inattentional blindness at a systems level in certain kinds of work. For example, organisations like the police can regulate the use of technology while driving.

### Limitations and future research directions

The findings of our survey provide crucial insight into the beliefs and experiences of different legal populations regarding inattentional blindness. However, some limitations of the survey should be noted, as they illuminate important avenues for further research.

First, participants were only able to answer “yes” or “no” as to whether they believed the individual in each legal scenario noticed the unexpected event. This response format prevents direct comparisons with the percentage of participants who actually noticed the events in the original research studies. Our choice of a binary response format was influenced by two key considerations. First, it aims to enhance ecological validity by emulating the type of yes/no decisions that legal populations would make in real-world cases disputing visual detection (e.g., reaching criminal verdicts and decisions of negligence in civil cases). For example, Boston police officer Kenny Conley was charged with perjury and obstruction of justice because jurors did not believe that he failed to notice an assault right in front of him while he was pursuing a suspect [68]. Jurors had to definitively reject the notion that Conley failed to notice the unexpected assault, and this definitive belief was directly tied to the legal decision-making (as to whether he perjured himself). Second, the response format mirrors the approach in established visual metacognition research [12, 15, 46], where participants are asked in retrospect whether they believe they would have noticed the unexpected event/change being described to them.

It would be fruitful for researchers looking to capture beliefs about inattentional blindness and visual detection in legal contexts to develop experiments where the beliefs can be directly compared back to the experimental research. For example, participants could instead be asked “*out of 100 people*, *how many people do you believe would notice the unexpected event*?” This would be a logical next step in this research area to determine whether legal professionals do genuinely overestimate visual detection, as has been hinted at in the research conducted with student populations [12, 13]. Additionally, as the research base on the factors that affect inattentional blindness and visual detection expands, it would be good to consider how beliefs held by different populations align with emerging research findings. For example, recent research has revealed that rapid motion of the unexpected event can reduce inattentional blindness [69].

Another limitation of our survey is that our legal scenarios were very brief. To make the vignettes simple and to minimise attrition among lawyers and police officers, we provided participants with only basic information in each scenario. With additional contextual information, it is possible that these beliefs in noticing may shift. For future research, it would be useful to include longer legal vignettes that mimic the type of information that would be provided in a legal case [17] or to show stimuli from original inattentional blindness studies and ask participants to make retrospective judgments about noticing [13], to provide a more complete picture of the context.

It is also important to consider the structure of the survey and the order of the questions when interpreting the results. To avoid priming participants that the survey was looking at inattentional blindness, the survey began with the scenario questions about noticing an unexpected event and factors that affect noticing an unexpected event. The term inattentional blindness was never mentioned in these scenarios and was mentioned for the first time after these questions had been answered. Due to this structure, we cannot rule out the possibility that the scenario and factor questions affected responses to subsequent questions. For example, participants who were unfamiliar with inattentional blindness may have drawn on information from the scenario questions when answering the later questions (e.g., about situations in which inattentional blindness might occur). However, given that participants who reported being familiar with inattentional blindness were often able to provide additional information beyond that given in the survey (e.g., by describing studies of inattentional blindness, or stating how they knew about the phenomenon), we do not have a strong reason to believe the structure of the survey significantly affected the conclusions that can be drawn about familiarity and anecdotal experience with inattentional blindness.

Finally, it should be noted that our participants consisted of Australian legal professionals and community members/psychology students residing in Australia. It is possible that the beliefs obtained in our survey may not generalise to other countries. It would be useful for other researchers to survey legal professionals working in other countries, to see if the beliefs about inattentional blindness and visual detection we have obtained are consistent across different contexts and legal frameworks.

## Conclusion

Inattentional blindness is a failure of visual awareness that is counterintuitive [7] and surprising [8, 9]. Given that inattentional blindness can and does occur in situations that have legal ramifications [17, 22], it is important to increase our understanding of the beliefs held about inattentional blindness by different populations that operate within the legal system—such as police officers and lawyers. The preliminary findings from our survey suggest that legal populations may commonly believe individuals will notice unexpected legal events. This is even though knowledge about inattentional blindness is not uncommon, and people can reflect on their own experiences of inattentional blindness. Our findings highlight ripe opportunities for training and educating legal professionals about inattentional blindness and visual detection. Further research is required to understand exactly how these visual metacognitive beliefs map directly on to the actual rates of inattentional blindness in research studies, as well as how beliefs about inattentional blindness can subsequently affect actual behaviours and decision-making in legal settings.
